# Bacterial internalization is required to trigger NIK-dependent NF-κB activation in response to the bacterial type three secretion system

**DOI:** 10.1371/journal.pone.0171406

**Published:** 2017-02-06

**Authors:** Miles C. Duncan, Natalia G. Herrera, Kevin S. Johnson, Joanne N. Engel, Victoria Auerbuch

**Affiliations:** 1 Department of Microbiology and Environmental Toxicology, University of California Santa Cruz, Santa Cruz, California, United States of America; 2 Department of Microbiology and Immunology, University of California San Francisco, San Francisco, California, United States of America; 3 Department of Medicine, University of California San Francisco, San Francisco, California, United States of America; University of the Pacific, UNITED STATES

## Abstract

Infection of human cells with *Yersinia pseudotuberculosis* expressing a functional type III secretion system (T3SS) leads to activation of host NF-κB. We show that the *Yersinia* T3SS activates distinct NF-κB pathways dependent upon bacterial subcellular localization. We found that wildtype *Yersinia* able to remain extracellular triggered NF-κB activation independently of the non-canonical NF-κB kinase NIK in HEK293T cells. In contrast, *Yersinia* lacking the actin-targeting effectors YopEHO, which become internalized into host cells, induce a NIK-dependent response and nuclear entry of the non-canonical NF-κB subunit p52. Blocking actin polymerization and uptake of effector mutant bacteria using cytochalasin D shifted the host NF-κB response from NIK-independent to primarily NIK-dependent. We observed similar results using *Pseudomonas aeruginosa*, which expresses a related T3SS and the actin-targeting effector ExoT. As the NF-κB response of HEK293T cells to effectorless *Yersinia* has been used both as a screening tool for chemical inhibitors of the T3SS and for bacterial forward genetic screens, a better understanding of this response is important for tool optimization and interpretation.

## Introduction

Bacterial pathogens and the mammalian innate immune system have co-evolved over millennia to recognize and combat one another. During infection, bacteria use virulence factors to survive and subvert host defenses, while the immune system employs pattern recognition receptors (PRRs) to identify and respond to these and other danger signals. PRRs, including Toll-like receptors (TLRs), NOD1 and NOD2, and nucleotide-binding domain leucine-rich repeat-containing receptors (NLRs), sense pathogen-associated molecular patterns (PAMPs) like bacterial flagellin or lipopolysaccharide to activate appropriate inflammatory responses. These PRRs occupy different cellular locations, allowing infected cells to recognize cytosolic, endosomal, and extracellular insults [1].

One common virulence factor, which is also recognized as a PAMP, is the bacterial type III secretion system (T3SS) [2]. The T3SS is a needle-like apparatus employed by dozens of bacterial pathogens to inject effector proteins into target host cells. Once inside, translocated effectors carry out species-specific functions, including dampening of immune signaling and prevention of phagocytosis by blocking actin polymerization. Effector translocation requires the translocator proteins YopB and YopD in pathogenic *Yersinia* (PopB and PopD in *Pseudomonas aeruginosa*), which are thought to insert in the host cell membrane and form a conduit through which effector proteins are injected into the host cytosol [3]. YopB and YopD were previously found to be important for the ability of *Y*. *pseudotuberculosis* to induce NF-κB activation in human cells [4–6] suggesting that these proteins stimulate host cell signaling in addition to enabling translocation of effector proteins [7]. This translocator-dependent NF-κB activation was independent of host TLRs, Nod1 and Nod2, and the caspase-1 inflammasome [4]. Furthermore, T3SS effectors were dispensable for this response. In fact, the T3SS effector protein YopJ is known to dampen NF-κB signaling [8].

NF-κB is a family of inducible mammalian transcription factors important for expression of inflammatory, developmental, and survival genes [9]. The canonical NF-κB pathway controls gene expression through activity of the RelA/p50 heterodimer. This pathway is primarily involved in transient proinflammatory gene expression, while non-canonical NF-κB results in a slower, persistent response [10]. Unlike canonical NF-κB, the non-canonical pathway is generally not associated with innate immune responses and results in nuclear translocation of heterodimerized RelB and p52 [11]. This alternative pathway requires NF-κB inducing kinase (NIK), a central hub that integrates signals from various membrane receptors including B-cell activating factor receptor (BAFFR). Non-canonical NF-κB signaling is essential for regulating bone metabolism, dendritic cell activation, B-cell survival, and lymphoid organogenesis [11]. The molecular implications of non-canonical activation are diverse and dependent on cell type. For instance, B cells produce anti-apoptotic genes like *bcl-x* and *bcl-2* in response to BAFF ligand, which are important for cell survival and maturation. In addition, cells with the lymphotoxin beta receptor respond to their cognate ligand by producing CCL21, CCL19, CXCL13, and CXCL12, chemokines essential for lymphoid development [12].

Here we examine the nature of the NF-κB response to *Y*. *pseudotuberculosis* and *P*. *aeruginosa* expressing or lacking T3SS effectors in human embryonic kidney HEK293T cells, commonly used as a model system for studying innate immunity to bacterial pathogens [13–15]. We find that, surprisingly, HEK293T cells launch a non-canonical NF-κB response to *Yersinia* or *Pseudomonas* lacking actin-targeting T3SS effectors but expressing an otherwise functional T3SS.

## Materials and methods

### Bacterial strains and cell lines

The *Y*. *pseudotuberculosis* IP2666 and *P*. *aeruginosa* PA103 strains used in this study are listed in S1 Table. The *Y*. *pseudotuberculosis* Δ*yopH* and Δ*yopO* deletions in the IP2666 background were constructed as previously described [16], using primer pairs designed using Primer 3 software (http://fokker.wi.mit.edu/primer3/input.htm): F5’yopO (CGGTGAATGGGGATACAAAG), R5’yopO (TAGGGGGCACTTGTCACATCCCCATGATTTTCACGCTTTT), F3’yopO (AAAAGCGTGAAAATCATGGGGATGTGACAAGTGCCCCCTA), R3’yopO (TGGAGAAATGGCAATCAGGT), F5’yopH (CGCCAGACATTCACGACTAA), R5’yopH (TTGATTGGCAAAGTGGTTTTTAATAATAGGTGAGCCGTGT), F3’yopH (GGCTCACCTATTATTAAAAACCACTTTGCCAATCAAAGAA), R3’yopH (ACCACTGCTGGTCAGTCGAT).

HEK293T cells (ATCC) were maintained in Dulbecco’s modified Eagle’s medium (DMEM) supplemented with 10% fetal bovine serum (FBS) and 2 mM L-glutamine at 37°C in 5% CO_2_.

### Bacterial culture and infection conditions

*P*. *aeruginosa* were grown overnight in Luria broth (LB) at 37°C with shaking. On the day of infection, cultures were backdiluted to an OD_600_ of 0.2 into LB and incubated at 37°C with shaking for 3 hours. Cultures were normalized for OD_600_ and added to eukaryotic cells for infection.

*Y*. *pseudotuberculosis* cultures were grown overnight in 2xYT media at 26°C with shaking. On the day of infection, the cultures were backdiluted to an OD_600_ of 0.2 into low calcium media (2xYT with 20 mM sodium oxalate and 20 mM MgCl_2_) and incubated at 26°C with shaking for 1.5 hours. The cultures were then shifted to 37°C with shaking for 1.5 hrs. Cultures were normalized for OD_600_ and added to eukaryotic cells for infection.

### Western blots

To assess p52 and p50 nuclear translocation and NIK levels, HEK293T cells were plated at a density of 5x10^5^ cells per well of a 6-well plate and incubated overnight. The next day, the cells were infected with *Y*. *pseudotuberculosis* at a multiplicity of infection (MOI) of 10 or 25 for 4 hrs. The cells were resuspended in RIPA buffer and lysed by sonication at 40% power with four 10-second pulses and a 20 second rest on ice. For nuclear and cytoplasmic protein isolation, the Thermo Fisher NE-PER kit was used according to the manufacturer’s instructions with the exception of isolating nuclear proteins with sonication at 40% power with two 10-second pulses and 20 second rest on ice. Lysates were run on a 7.5% SDS-PAGE gels. Following semi-dry transfer or wet transfer (for visualization of NIK) to immobilon-P, samples were analyzed by Western blotting for p100/p52 (Cell Signaling), p105/p50 (Millipore), actin (Santa Cruz Biotechnology), laminB1 (Cell Signaling), tubulin (Cell Signaling), and NIK (Santa Cruz Biotechnology). Densitometric quantification of the bands was performed using Image Lab software (Bio-Rad).

### RNA interference

3x10^4^ HEK293Ts cells were plated in each well of a 24-well plate in 400 μl DMEM plus 10% FBS and incubated overnight. On day two, the cells were transfected with siRNA targeting NIK or scrambled control siRNA (siCON, Dharmacon) using Lipofectamine 2000 (Invitrogen) according to the manufacturer’s instructions. The following day the cells were transfected with an NF-κB luciferase reporter plasmid (Stratagene) using Lipofectamine 2000 (Invitrogen) according to the manufacturer’s instructions. On day four, the HEK293Ts were infected with *Y*. *pseudotuberculosis* at MOI 7. Following a four hour infection at 37°C/5% CO2, the medium was aspirated before adding a 1:1 Neolite–phosphate-buffered saline (PBS) solution. Plates were covered in foil, incubated for 5 min, and bioluminescence measured using an EnVision plate reader (PerkinElmer).

### Filtered supernatant experiment

HEK293T cells were plated and transfected with the NF-κB reporter plasmid as described above. Concurrently, the same number of HEK293T cells were plated in a separate 24-well plate, but not transfected. The non-transfected cells were infected as described above. Following a four-hour infection, the cell supernatants were filtered through a 0.22 μm filter, and this filtered supernatant (conditioned media) transferred to the transfected reporter HEK293T cells. As a positive control, some wells of transfected reporter cells were infected with *Y*. *pseudotuberculosis*. After four hours of incubation with the conditioned media, or infection, bioluminescence (NF-κB activation) was measured as described above.

### Invasion assay

12mm round coverslips were autoclaved and treated with sterile Poly-L lysine (Sigma) diluted 1:10 in water for 5 min. Coverslips were washed with sterile water and allowed to dry for at least 2 hours before use. 2.6x10^5^ HEK293T cells were plated in each well of a 96-well plate in 400 μl DMEM plus 10% FBS and incubated overnight. The following day, the HEK293T cells were infected at MOI 7. After 30 min, cell media was aspirated and replaced with fresh media to remove non-attached cells. To end the infection, after two total hours the cells were fixed with 4% formaldehyde for 10 min and incubated with an anti-*Yersinia* antibody (generously provided by R. Isberg) followed by an anti-rabbit Alexa-fluor 594 secondary antibody (Life Technologies). The HEK293T cells were then permeabilized with ice-cold methanol for 10 sec, incubated with the anti-*Yersinia* antibody followed by an anti-rabbit FITC secondary antibody (Santa Cruz Biotechnology) and Hoechst diluted 1:10,000 in PBS, mounted using ProLong Gold mounting media (Life Technologies), and imaged using a Leica SP5 Confocal Microscope. Approximately 300 bacteria were counted per strain for each of three biological replicates.

### Cytotoxicity assay

4.2x10^4^ HEK293T cells were plated in each well of a 96-well plate in 100 μl DMEM plus 10% FBS and incubated overnight. The following day the cells were infected in triplicate at MOI 7. The cells were then incubated at 37°C/5% CO2 for 4 or 24 hours. For the 24-hour time point, chloramphenicol was added four hours post-inoculation. Following infection, the supernatant was transferred to microcentrifuge tubes and centrifuged to pellet cellular debris. For full cell lysis, 3 wells were flash frozen. 50 μl of the supernatant was then transferred to a new 96-well well plate, and incubated for 30 minutes with 50 μl of substrate mix (CytoTox 96 Non-Radioactive Cytotoxicity Assay, Promega). Following the incubation, 50 μl of stop solution was added to each well, and the absorbance measured at 490nm. The fully lysed wells were averaged and percent LDH release was calculated for each infection condition.

### Statistical analysis

Unless stated otherwise, bar charts display a mean ± SEM from at least 3 independent biological experiments. P values were determined by one-way ANOVA with Tukey’s post-hoc test comparing all strains or conditions. Student’s t-test was also used for additional analysis to compare NF-κB activation in siNIK versus siCON-treated conditions for a given bacterial strain. All statistical analysis was done using KaleidaGraph (Synergy Software).

## Results

### The *Y*. *pseudotuberculosis* T3SS triggers a NIK-dependent NF-κB response

**Fig 1:**
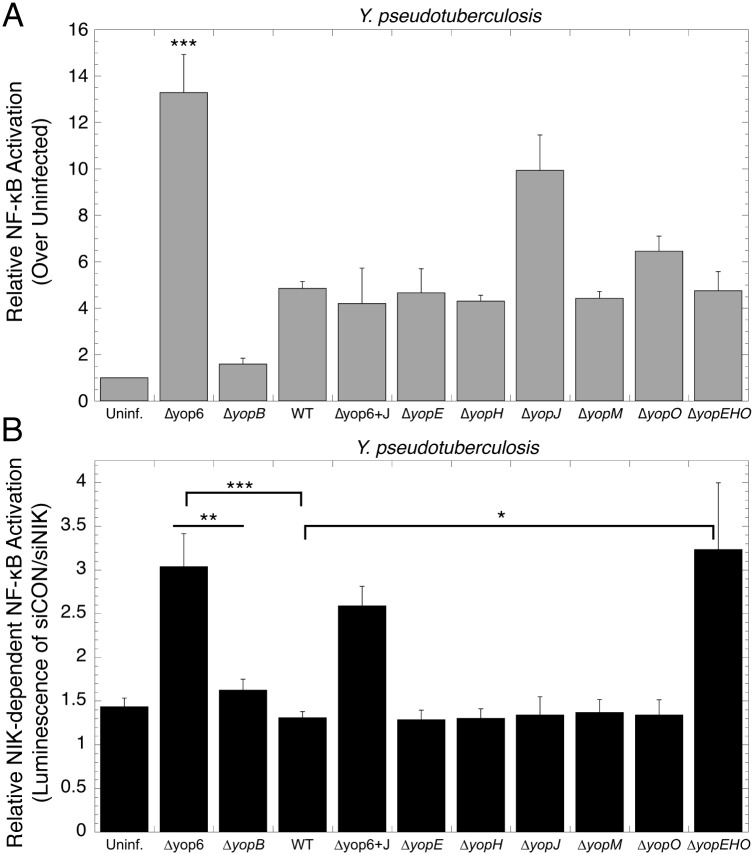
*Yersinia* lacking actin-targeting effectors activate NIK-dependent NF-κB activation. **(A)** NF-κB luciferase reporter HEK293T cells were infected with WT and mutant *Yersinia* for four hours and luminescence measured. **(B)** The same experiment as in A was repeated, except NIK expression was knocked down using siRNA, or control siRNA was used. Data is expressed as NF-κB levels in control siRNA-treated cells divided by NF-κB levels of cells treated with siRNA against NIK. Higher ratios indicate more dependence on NIK. The average of at least three independent biological replicates ± standard error of the mean (SEM) is shown. *p < 0.05, **p < 0.005, ***p < 0.0005, as determined by one-way ANOVA with Tukey’s HSD post-hoc test.

To begin to characterize the HEK293T cell NF-κB response to *Yersinia*, we analyzed activity of an NF-κB responsive luciferase reporter gene in cells infected with *Y*. *pseudotuberculosis* T3SS mutants [17]. As previously shown [4], a mutant lacking the YopHEMOJT T3SS effector proteins (Δyop6) induced significantly higher levels of NF-κB activity than the WT strain encoding the YopHEMOJ effectors or a mutant unable to form pores in host cell membranes to deliver Yops (Δ*yopB*; Fig 1A). Loss of YopJ, an acetyltransferase known to dampen NF-κB activation [18, 19], led to increased NF-κB activation, although this was not statistically significant. However, a Δyop6 strain complemented with a plasmid encoding YopJ triggered less overall NF-κB activity than the parental Δyop6 strain (Fig 1A).

To determine the type of NF-κB pathway activated by *Yersinia*, we used RNA interference to deplete cellular levels of the non-canonical NF-κB kinase NIK prior to transfection with NF-κB luciferase reporter and infection with *Yersinia*. A representative NIK immunoblot shows a two-fold depletion of NIK levels upon RNA interference (S1 Fig), as typically seen in other studies [20–22]. Upon NIK knockdown, the Δyop6 strain triggered three-fold less NF-κB activity than in HEK293Ts treated with control siRNA (p = 0.003, Student’s t-test, Δyop6-siRNA control versus Δyop6-siNIK). This result suggests that the NF-κB response to the Δyop6 strain at four hours post-infection is primarily NIK-dependent, as at least 63% of the overall NF-κB response measured is dependent on NIK.

WT *Y*. *pseudotuberculosis*, which triggered less overall NF-κB activity compared to the Δyop6 strain, activated NF-κB independently of NIK (Fig 1). Expression of YopJ was not sufficient to shift the response from NIK-independent to NIK-dependent, as the Δyop6+pYopJ strain induced NF-κB activation in a manner that was not statistically different from the Δyop6 parental strain during NIK knockdown (Fig 1B). Taken together, these data confirm that YopJ reduces overall NF-κB activation, yet its presence or absence does not impact the NIK-dependency of the NF-κB response. Furthermore, these results suggest that Yop effectors other than YopJ alter the NIK-dependency of the NF-κB response.

### Yop effectors that target the actin cytoskeleton shift the NIK-dependency of the host cell NF-κB response

We sought to determine whether a Yop effector other than YopJ was responsible for influencing the nature of the NF-κB response to *Yersinia*. We infected HEK293T cells with single-effector deletion mutants lacking Yops E, H, J, M, or O, and tested their NF-κB response (Fig 1). All five single deletion strains triggered a primarily NIK-independent NF-κB response at a similar level to WT *Y*. *pseudotuberculosis* (Fig 1B), indicating that no single effector was responsible for shifting the NF-κB response from largely NIK-dependent to NIK-independent.

As several Yop effectors target the actin cytoskeleton, we tested the ability of a *Y*. *pseudotuberculosis* strain lacking the actin cytoskeleton targeting effectors E, H, and O, but expressing Yops J and M, to activate NF-κB in HEK293T cells. The Δ*yopEHO* mutant induced NF-κB to the same level as WT *Y*. *pseudotuberculosis* (Fig 1A), as YopJ is expressed, but required NIK to activate NF-κB (p = 0.02, Student’s t-test, Δ*yopEHO*-siRNA control versus Δ *yopEHO*-siNIK; Fig 1B) similar to the Δyop6 strain. This suggests that the actin-targeting effector Yops E, H, and O are collectively required to alter the NIK-dependency of the NF-κB response, and that Yops J and M are dispensable for this effect.

### The *Pseudomonas aeruginosa* T3SS triggers a primarily NIK-dependent NF-κB response

To determine whether the NIK-dependent NF-κB response is unique to *Y*. *pseudotuberculosis*, or could be observed during infection with other T3SS-expressing pathogens, we tested the immune response to *Pseudomonas aeruginosa*. We compared PA103 strains lacking the T3SS effectors ExoU and ExoT (Δ*exoUT*), containing a nonfunctional T3SS (Δ*popBD*), or containing only the actin-targeting effector ExoT (Δ*exoU)*. A recent report identified PemA and PemB as putative *Pseudomonas* effectors, and these may be present [23]. WT *P*. *aeruginosa* could not be used in this assay as its phospholipase ExoU rapidly lysed host cells before the NF-κB response could be measured (data not shown).

**Fig 2:**
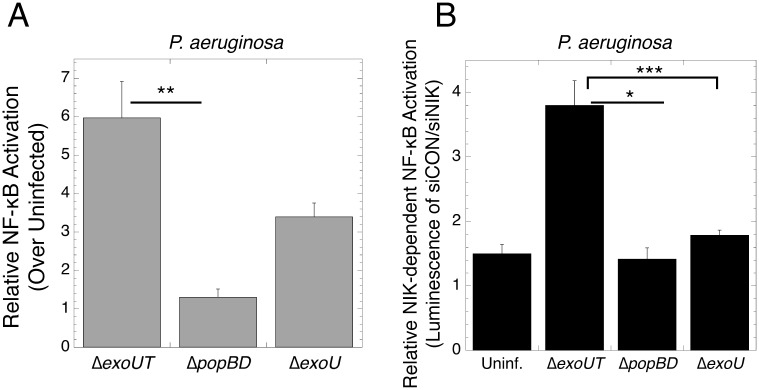
*Pseudomonas* lacking actin-targeting effectors trigger NIK-dependent NF-κB. **(A)** NF-κB luciferase reporter HEK293T cells were infected with various *Pseudomonas* strains for four hours and luminescence measured. **(B)** The same experiment as in A was repeated in the presence of control siRNA or siRNA against NIK. The average of at least three independent biological replicates ± SEM is shown. *p < 0.05, **p < 0.005, ***p < 0.0005, as determined by one-way ANOVA with Tukey’s HSD post-hoc test.

*P*. *aeruginosa* Δ*exoUT* caused 4.5-fold more NF-κB activation than the Δ*popBD* strain (p = 0.005, ANOVA with Tukey’s HSD post-hoc), while the Δ*exoU* strain did not induce a significant response (p = 0.29, ANOVA Tukey’s HSD post-hoc, Fig 2A). Like the Δyop6 *Y*. *pseudotuberculosis* strain, Δ*exoUT* triggered NF-κB that was 73% NIK-dependent in contrast to the Δ*popBD* or Δ*exoU P*. *aeruginosa* strains (Fig 2B). This indicates that *P*. *aeruginosa* and *Y*. *pseudotuberculosis* strains lacking actin-targeting effectors induce a largely NIK-dependent NF-κB pathway, and that translocation of actin-modulating effectors into host cells alters the NIK-dependency of the NF-κB response to infection with T3SS-positive bacteria.

### The *Y*. *pseudotuberculosis* T3SS triggers nuclear localization of the non-canonical subunit, p52

**Fig 3:**
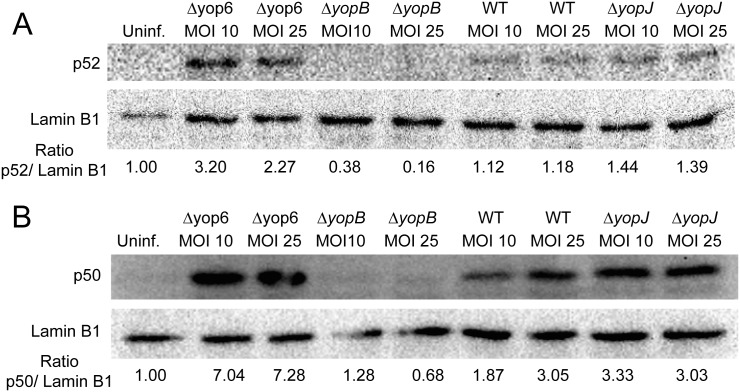
*Yersinia* lacking actin-targeting effectors activate nuclear translocation of NF-κB subunits p52 and p50. HEK293T cells were infected with various *Y*. *pseudotuberculosis* mutants for four hours and nuclear extracts were isolated. **(A)** p52 and **(B)** p50 levels were measured by immunoblot and compared to nuclear Lamin B1 levels. Representative images are shown.

In unstimulated cells, NIK protein levels are kept low by the activity of a destruction complex, as activation of the non-canonical NF-κB pathway requires NIK protein levels to accumulate [24, 25]. This complex, comprised of TRAF3, TRAF2, and cIAP1/2, continuously degrades NIK until the complex is recruited to one of several ligand-bound membrane receptors [12]. The presence of NIK is essential for processing of the non-canonical NF-κB precursor, p100, into p52 and induction of nuclear translocation of the non-canonical NF-κB heterodimer, RelB/p52 [24, 25]. To assess the involvement of p52 in the HEK293T cell response to *Yersinia*, we analyzed p52 nuclear entry in infected HEK293T cells via western blot (Fig 3A). The Δyop6 strain induced greater p52 accumulation in comparison to WT, the ΔyopJ strain, and the ΔyopB strain as well as uninfected HEK293T cells, although some p52 was detected in the nucleus of cells infected with WT and the Δ*yopJ* mutant. Collectively these data show that *Yersinia* lacking actin-targeting effectors induces greater p52 nuclear entry and NIK-dependent NF-κB activation than *Yersinia* expressing these effectors.

### The *Y*. *pseudotuberculosis* T3SS induces nuclear localization of the canonical NF-κB subunit, p50

To assess the canonical NF-κB response to *Yersinia*, we infected HEK293T cells and measured nuclear localization of p50 (Fig 3B). Interestingly, the Δyop6 strain induced approximately two-fold more nuclear p50 than both WT and the Δ*yopJ* strain. These data suggest that although the majority of the NF-κB response to *Yersinia* lacking actin-targeting effectors is NIK-dependent, both p50 and p52 subunits enter the nucleus following HEK293T infection with Δyop6.

### Actin-targeting effectors prevent *Y*. *pseudotuberculosis* invasion of HEK293T cells

**Fig 4:**
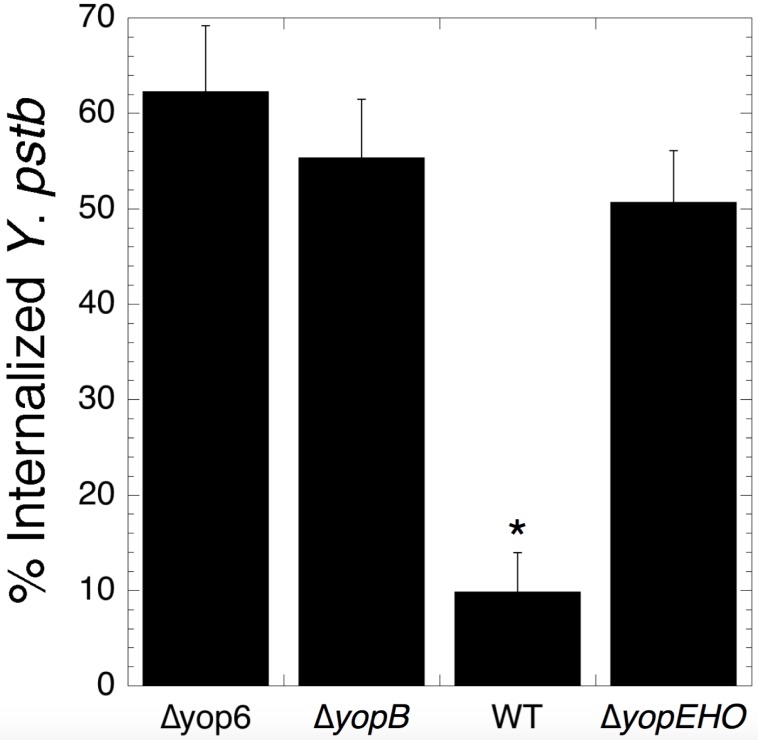
*Yersinia* mutants that cannot target the actin-cytoskeleton are internalized into HEK293T cells. HEK293T cells were infected with WT and mutant *Yersinia* for two hours and infected cells visualized by confocal microscopy. The average percent of internalized bacteria of three independent biological replicates ± SEM is shown. *p < 0.005, as determined by one-way ANOVA with Tukey’s HSD post-hoc test.

To further understand how the T3SS activates a predominant NIK-dependent NF-κB response in the absence of YopEHO, we investigated the ability of our *Y*. *pseudotuberculosis* strains to invade HEK293T cells (Fig 4, S2 Fig). WT *Y*. *pseudotuberculosis* remained largely extracellular. The Δyop6 strain invaded at the highest rate, with 62% of bacteria internalized two hours post-inoculation. The Δ*yopEHO* strain and the T3SS-deficient Δ*yopB* strain were also unable to prevent internalization and displayed 51 and 55% invasion, respectively. These results suggest that internalization is necessary but not sufficient for NIK-dependent NF-κB activation, as the Δyop6, Δ*yopEHO*, and Δ*yopB* strains invaded, but only the Δyop6 and Δ*yopEHO* strains, containing a functional T3SS, triggered a primarily NIK-dependent NF-κB response. This differential localization did not lead to differences in host cell survival, as infection with the WT, Δyop6, and Δ*yopB* strains did not cause significant LDH release compared to uninfected controls (S4 Fig).

### Extracellular bacteria do not activate NIK-dependent NF-κB

**Fig 5:**
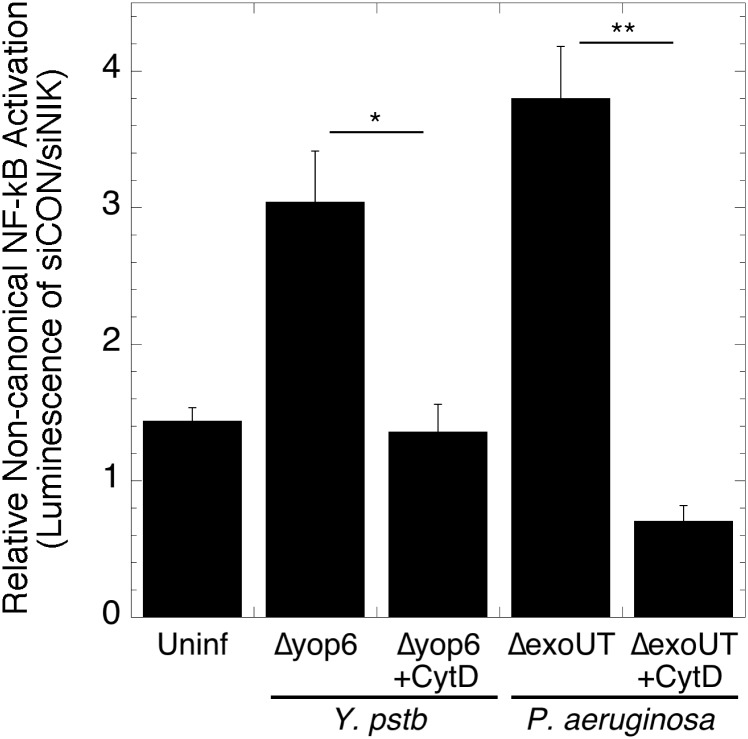
Intracellular and extracellular T3SS-positive bacteria vary in their dependency on NIK for NF-κB activation. NF-κB luciferase reporter HEK293T cells were treated with control siRNA or siRNA against NIK, infected with *Yersinia* or *Pseudomonas* for four hours in the presence or absence of the actin disrupting toxin cytochalasin D, and luminescence measured. The average of at least three independent biological replicates is shown ± SEM. *p < 0.03, **p < 0.004, as determined by one-way ANOVA with Tukey’s post-hoc test.

We hypothesized that location of T3SS activity, irrespective of effector presence, was the critical factor determining the type of NF-κB response. To test this, we pretreated HEK293T cells with the actin depolymerizing fungal toxin cytochalasin D to prevent bacterial internalization, and quantified NF-κB NIK dependency. Cytochalasin D pre-treatment of HEK293T cells infected with either *Y*. *pseudotuberculosis* Δyop6 or *P*. *aeruginosa* Δ*exoUT* led to loss of dependence on NIK for NF-κB activation (Fig 5). Furthermore, pre-treatment of infected cells with the Rac1 inhibitor NSC23766 [26] reduced Δyop6 bacterial uptake and NIK-dependent NF-κB activation by ~1.5 fold (S3 Fig). Taken together, these results suggest *Y*. *pseudotuberculosis* and *P*. *aeruginosa* must be internalized and possess a functional T3SS to activate NIK-dependent NF-κB.

### Conditioned media from *Y*. *pseudotuberculosis* infected cells does not stimulate NF-κB in naïve HEK293T cells

**Fig 6:**
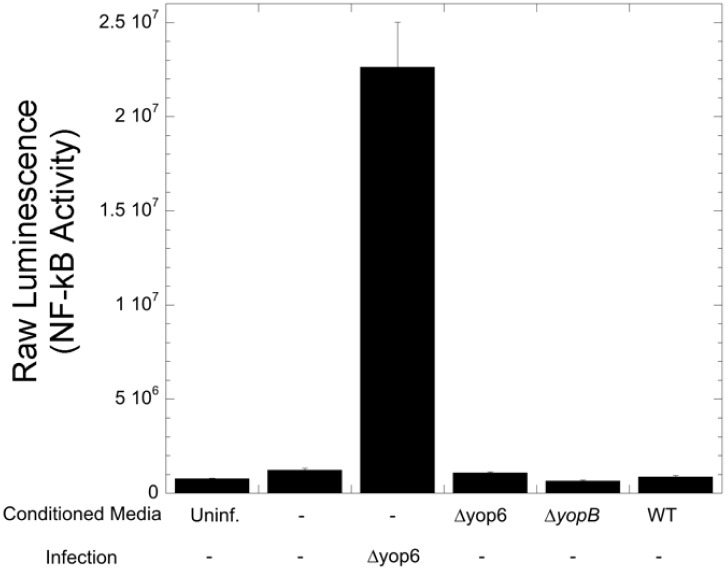
Transfer of conditioned media from *Yersinia* infected cells does not activate NF-κB in naïve cells. HEK293T cells were infected for four hours with various *Yersinia* strains, the supernatant filtered and transferred to uninfected HEK293T cells expressing an NF-κB luciferase reporter, and NF-κB activity measured after four hours. In parallel, as a positive control, HEK293T reporter cells were infected with *Y*. *pseudotuberculosis* Δyop6 and bioluminescence measured after four hours. A representative experiment of three independent replicates is shown.

In order to determine whether a diffusible molecule is involved in the ability of *Yersinia* to trigger NF-κB activation, we tested the ability of the supernatant of infected HEK293T cells to trigger NF-κB activation in naïve HEK293T NF-κB reporter cells (Fig 6). While infecting HEK293T cells with *Y*. *pseudotuberculosis* Δyop6 induced NF-κB activation as expected, incubating these cells with filtered supernatant from infected cells did not trigger NF-κB. These data suggest that *Yersinia* does not induce NF-κB activation in HEK293T cells dependent upon a diffusible molecule.

## Discussion

We used RNA interference to elucidate the host NF-κB response to the bacterial T3SS. T3SS-positive extracellular *Y*. *pseudotuberculosis* and *P*. *aeruginosa* triggered NF-κB activation in a NIK-independent manner, indicative of the canonical NF-κB pathway. In contrast, internalized T3SS-positive *Y pseudotuberculosis* and *P*. *aeruginosa* induced NF-κB activation in a largely NIK-dependent manner, indicative of the non-canonical NF-κB pathway. These data suggest that T3SS-expressing bacteria may trigger distinct signaling pathways following engagement of the plasma membrane versus the vacuolar membrane.

We found that internalized *Y*. *pseudotuberculosis* and *P*. *aeruginosa* induce a distinct NIK-dependent NF-κB pathway. This non-canonical NF-κB activation was dependent on a functional T3SS, as pore formation mutants (Δ*yopB* and Δ*popBD*) were internalized but did not induce NF-κB. If cytochalasin D was used to force bacteria expressing an active T3SS but lacking actin-targeting effectors to remain extracellular, the bacteria no longer activated NF-κB in a NIK-dependent manner. Cytochalasin D still allowed 60% of normal Yop translocation, suggesting it does not completely block translocation of T3SS cargo into the host cytosol ([26], unpublished data). In addition, the Rac1 inhibitor NSC23766, which does not have an effect on translocation or pore formation [26], also decreased bacterial uptake and NF-κB NIK-dependency. Therefore, non-canonical NF-κB signaling may be a mechanism to recognize T3SS activity in internalized bacteria.

The non-canonical NF-κB pathway is typically activated by a small set of membrane receptors and their cognate ligands, such as BAFF, CD40 and LTβ [10]. We have not yet identified the receptor responsible for T3SS-induced non-canonical NF-κB activation. A subset of ligands that trigger the non-canonical NF-κB pathway are secreted molecules such as BAFF [10, 12]. However, our data suggests that NF-κB activation triggered by *Yersinia* lacking actin-targeting effectors does not involve a diffusible molecule released from infected cells. This suggests that *Yersinia* lacking actin targeting effectors instead triggers NF-κB directly in infected cells or in bystander cells through modulation of a membrane-bound receptor agonist.

Previous studies have elucidated independent mechanisms by which host cells recognize the T3SS. There is evidence that translocation of T3SS needle and inner rod subunits through the T3SS into host cells leads to inflammasome activation via NAIP PRRs [15, 27–30]. In addition, *Yersinia* YopE and YopT were recently shown to trigger pyrin inflammasome activation through their impact on RhoA [31]. Furthermore, Keestra *et al*. demonstrated that the downstream activities of a *Salmonella* Rho GTPase-activating effector could be sensed by Nod1, leading to induction of NF-κB and MAPK pathways [32]. While we demonstrate here that actin cytoskeletal-targeting effectors alter the nature of the NF-κB response to T3SS-positive *Yersinia*, our previous data showed that the NF-κB response to the effectorless *Y*. *pseudotuberculosis* Δyop6 strain was independent of Nod1 and Nod2 [4]. Our findings suggest that a distinct host pathway may sense a functional T3SS deployed by an internalized pathogen, leading to NIK-dependent NF-κB activation.

While it remains unclear what implications this host cell signaling response has on infection, it is highly relevant for utilization of the NF-κB response to the T3SS as a screening strategy for T3SS activity. Previously our lab has used the host NF-κB response to screen for small-molecule inhibitors of the T3SS [17] and in a forward-genetic screen for bacterial genes affecting the T3SS [16]. A better understanding the NF-κB response to the T3SS will facilitate optimization of the signal to noise ratio of these screens, development of appropriate secondary screens, and insight into how small molecules or specific bacterial genes impact the T3SS-host cell interaction.

## Supporting information

S1 Fig Representative immunoblot of NIK protein levels following RNA interference. RNA interference was used to deplete NIK levels and dampen the non-canonical NF-κB response in Fig 1B. NIK protein levels were measured by immunoblot and compared to actin in the presence of control or NIK-targeting small-interfering RNA. A representative image is shown.
(TIF)

S2 Fig *Yersinia* internalization into HEK293T cells. HEK293T cells were infected with wildtype or mutant *Y*. *pseudotuberculosis* and intracellular versus extracellular bacteria enumerated after two hours of infection. Red bacteria are extracellular (left panels) whereas bacteria stained both red and green are intracellular (middle panels). HEK293T nuclei are stained with DAPI (blue). Representative images are shown.
(TIF)

S3 Fig Effect of NSC3766 and cytochalasin D on *Yersinia* invasion and NF-κB activation. **(A)** HEK293T cells were infected for four hours with Δyop6 in the presence or absence of cytochalasin D or NSC3766 and intracellular versus extracellular bacteria enumerated as in S2 Fig. **(B)** HEK293T cells expressing the NF-κB luciferase reporter treated with control siRNA or siRNA against NIK were infected for four hours with wildtype or mutant *Y*. *pseudotuberculosis* in the presence or absence of NSC3766 and bioluminescence quantified. The average of three independent experiments ± SEM is shown.
(TIF)

S4 Fig *Yersinia* toxicity to HEK293T cells. HEK293T cells were infected with WT, Δyop6, or Δ*yopB Yersinia* for four (A) or 24 hours (B) and cytotoxicity measured by lactate dehydrogenase (LDH) release. The average of three independent experiments ± SEM is shown.
(TIF)

S1 Table Bacterial strains used in this study. (DOCX)
