# Supplementary figures and images for: Bacterial internalization is required to trigger NIK-dependent NF-κB activation in response to the bacterial type three secretion system
Source: PLoS One. 2017 Feb 6;12(2):e0171406. doi: 10.1371/journal.pone.0171406 (PMC5293232; doi:10.1371/journal.pone.0171406)

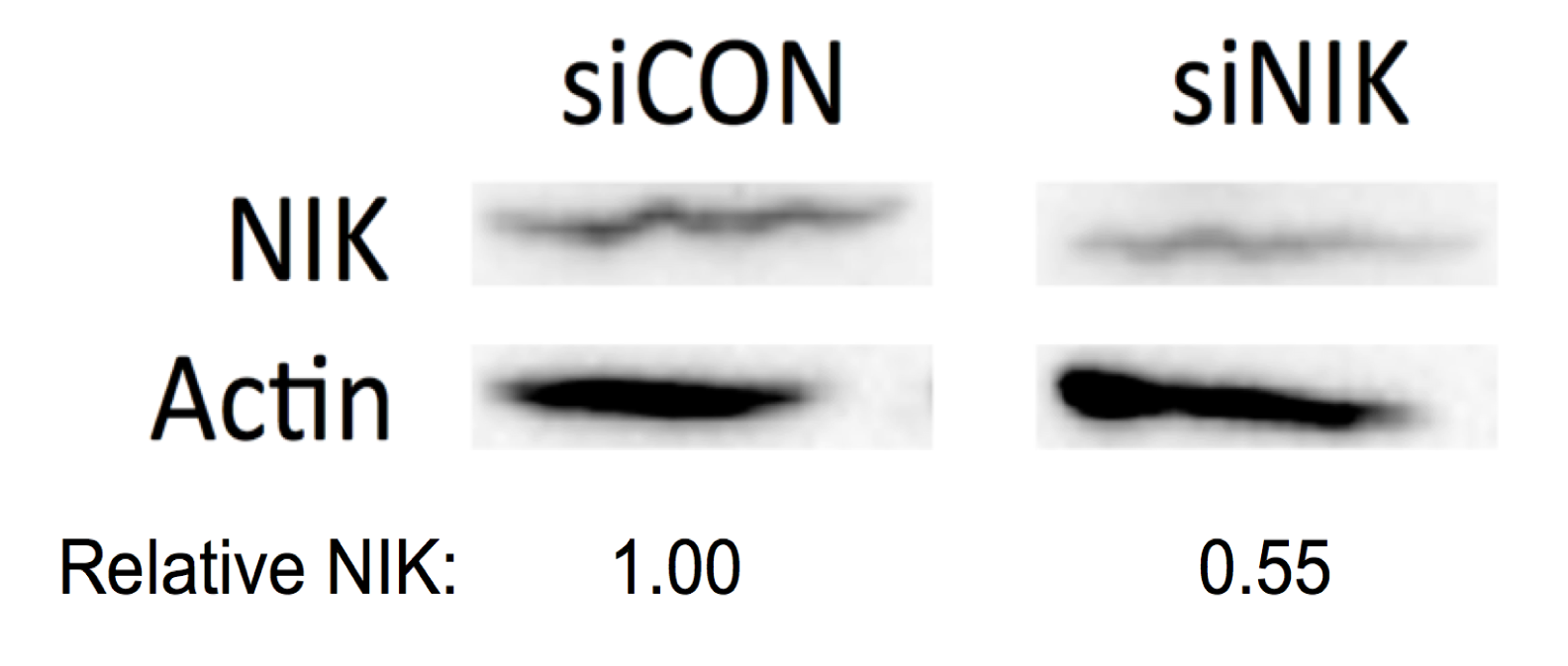

Supplement: S1 Fig — RNA interference was used to deplete NIK levels and dampen the non-canonical NF-κB response in Fig 1B. NIK protein levels were measured by immunoblot and compared to actin in the presence of control or NIK-targeting small-interfering RNA. A representative image is shown. (TIF) [file pone.0171406.s001.tif]

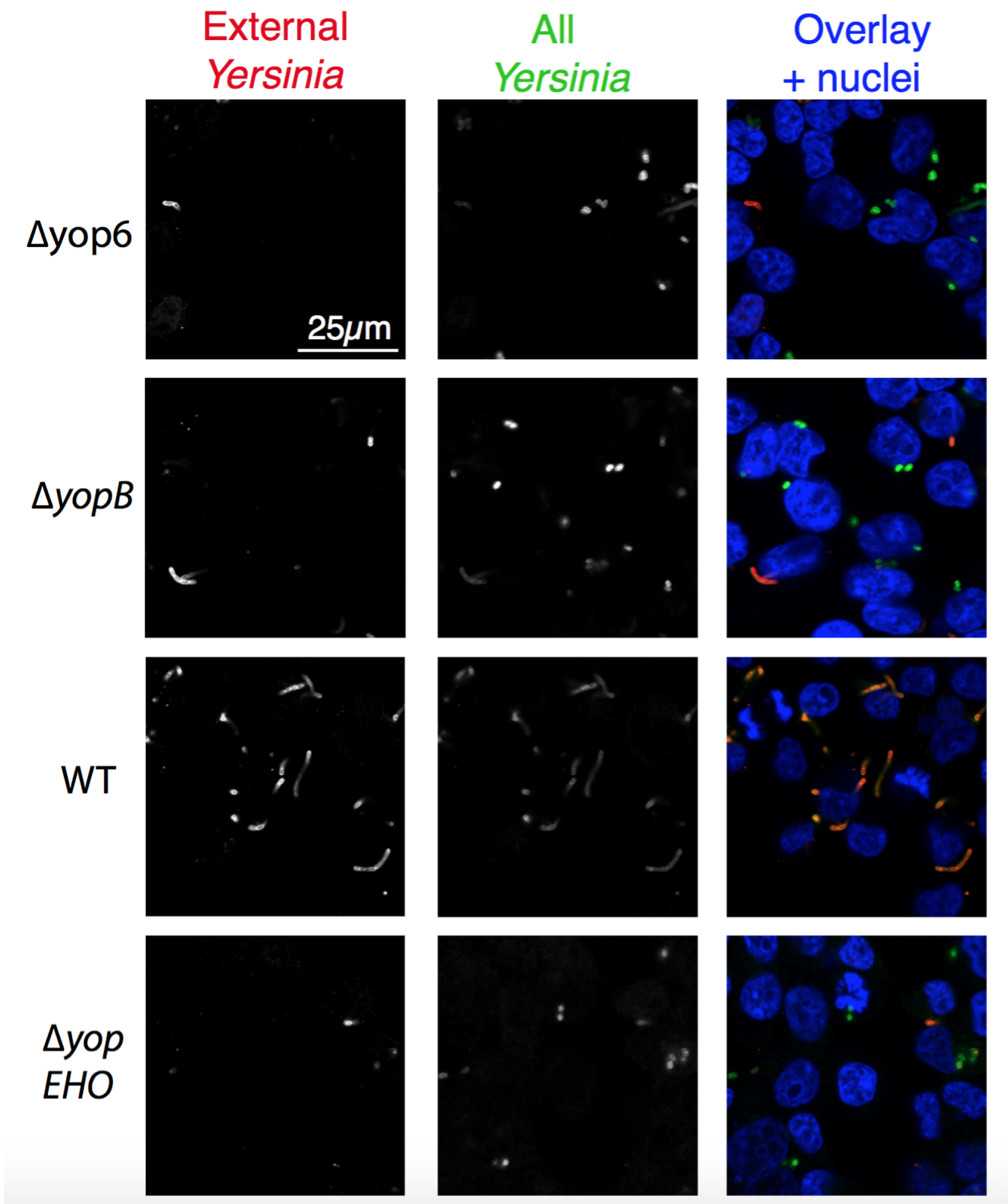

Supplement: S2 Fig — HEK293T cells were infected with wildtype or mutant Y. pseudotuberculosis and intracellular versus extracellular bacteria enumerated after two hours of infection. Red bacteria are extracellular (left panels) whereas bacteria stained both red and green are intracellular (middle panels). HEK293T nuclei are stained with DAPI (blue). Representative images are shown. (TIF) [file pone.0171406.s002.tif]

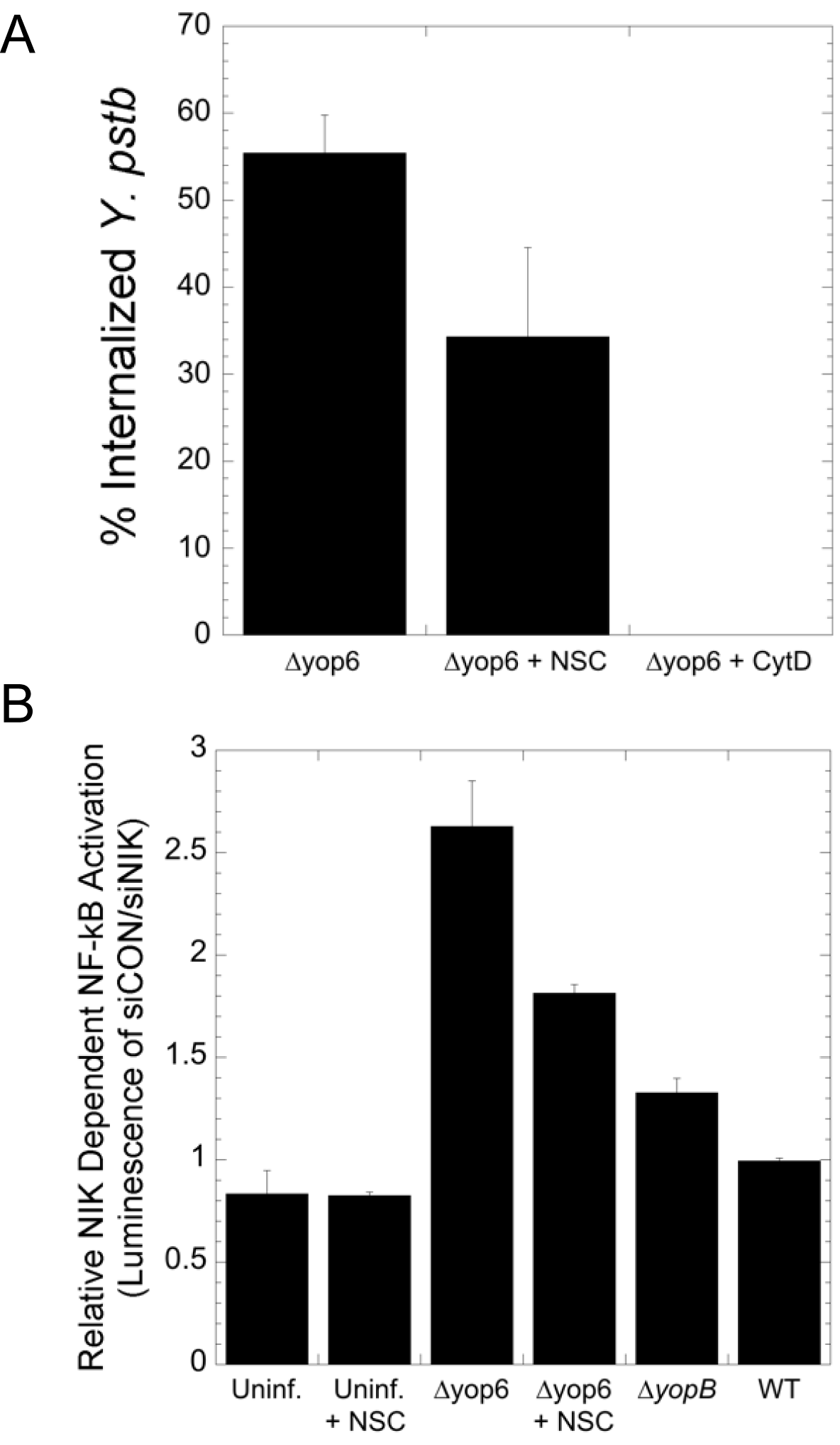

Supplement: S3 Fig — (A) HEK293T cells were infected for four hours with Δyop6 in the presence or absence of cytochalasin D or NSC3766 and intracellular versus extracellular bacteria enumerated as in S2 Fig. (B) HEK293T cells expressing the NF-κB luciferase reporter treated with control siRNA or siRNA against NIK were infected for four hours with wildtype or mutant Y. pseudotuberculosis in the presence or absence of NSC3766 and bioluminescence quantified. The average of three independent experiments ± SEM is shown. (TIF) [file pone.0171406.s003.tif]

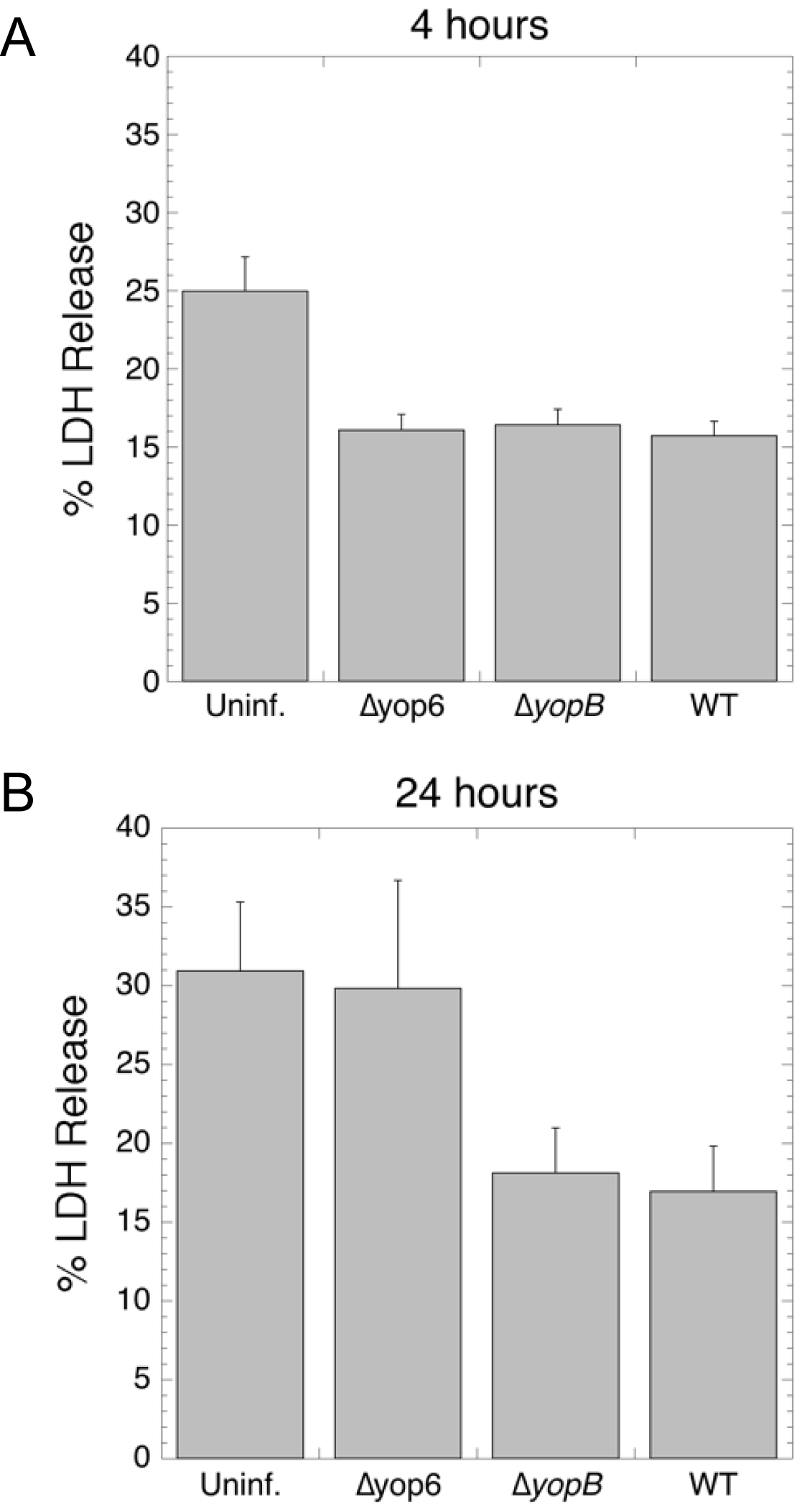

Supplement: S4 Fig — HEK293T cells were infected with WT, Δyop6, or ΔyopB Yersinia for four (A) or 24 hours (B) and cytotoxicity measured by lactate dehydrogenase (LDH) release. The average of three independent experiments ± SEM is shown. (TIF) [file pone.0171406.s004.tif]
