# Supplementary material for: Bacterial internalization is required to trigger NIK-dependent NF-κB activation in response to the bacterial type three secretion system
Source: PLoS One. 2017 Feb 6;12(2):e0171406. doi: 10.1371/journal.pone.0171406 (PMC5293232; doi:10.1371/journal.pone.0171406)
Supplement: S1 Table — (DOCX) [file pone.0171406.s005.docx]

| Strain | Description | References |
| --- | --- | --- |
| Wildtype | *Yersinia pseudotuberculosis* IP2666 (no YopT expression) | [1] |
| ∆yop6 | IP2666 ∆*yopHEMOJ* | [2] |
| ∆*yopB* | IP2666 ∆*yopB* | [2] |
| ∆yop6/∆*yopN* | IP2666 ∆*yopHEMOJN* | [2] |
| WT+YopH-Bla | IP2666 pMM83::yopM-bla fusion | [3] |
| ∆yop6+YopJ | IP2666 (MB202) | M. Bergman, unpublished |
| ∆*yopM* | IP2666 ∆*yopM* | [3] |
| ∆*yopJ* | IP2666 ∆*yopJ* | [3] |
| ∆*yopE* | IP2666 ∆*yopE* | [3] |
| ∆*yopH* | IP2666 ∆*yopH* | This work |
| ∆*yopO* | IP2666 ∆*yopO* | This work |
| ∆*yopEHO* | IP2666 ∆*yopEHO* (Contains YopJ and YopM) | This work |
|  |  |  |
| ∆*exoUT* | *Pseudomonas aeruginosa* PA103 ∆*exoUT* | [4] |
| ∆*exoU* | PA103 ∆*exoU* | [4] |
| ∆*popBD* | PA103 ∆*popBD* | [5] |

1. Bliska JB, Guan KL, Dixon JE, Falkow S. Tyrosine phosphate hydrolysis of host proteins by an essential *Yersinia* virulence determinant. Proc Natl Acad Sci U S A. 1991;88(4):1187-91. PubMed PMID: 1705028; PubMed Central PMCID: PMCPMC50982.

2. Auerbuch V, Golenbock DT, Isberg RR. Innate immune recognition of *Yersinia pseudotuberculosis* type III secretion. PLoS Pathog. 2009;5(12):e1000686. doi: 10.1371/journal.ppat.1000686. PubMed PMID: 19997504; PubMed Central PMCID: PMC2779593.

3. Adams W, Morgan J, Kwuan L, Auerbuch V. *Yersinia pseudotuberculosis* YopD mutants that genetically separate effector protein translocation from host membrane disruption. Mol Microbiol. 2015;96(4):764-78. doi: 10.1111/mmi.12970. PubMed PMID: 25684661.

4. Garrity-Ryan L, Kazmierczak B, Kowal R, Comolli J, Hauser A, Engel JN. The arginine finger domain of ExoT contributes to actin cytoskeleton disruption and inhibition of internalization of *Pseudomonas aeruginosa* by epithelial cells and macrophages. Infect Immun. 2000;68(12):7100-13. PubMed PMID: 11083836; PubMed Central PMCID: PMC97821.

5. Kang PJ, Hauser AR, Apodaca G, Fleiszig SM, Wiener-Kronish J, Mostov K, et al. Identification of *Pseudomonas aeruginosa* genes required for epithelial cell injury. Mol Microbiol. 1997;24(6):1249-62. PubMed PMID: 9218773.
